# *LARGE ROOT ANGLE1*, encoding OsPIN2, is involved in root system architecture in rice

**DOI:** 10.1093/jxb/erx427

**Published:** 2017-12-22

**Authors:** Lingling Wang, Mengxue Guo, Yong Li, Wenyuan Ruan, Xiaorong Mo, Zhongchang Wu, Craig J Sturrock, Hao Yu, Chungui Lu, Jinrong Peng, Chuanzao Mao

**Affiliations:** 1 State Key Laboratory of Plant Physiology and Biochemistry, College of Life Sciences, Zhejiang University, Hangzhou, China; 2 The Hounsfield Facility, School of Biosciences, University of Nottingham, Nottingham, UK; 3 Department of Biological Sciences and Temasek Life Sciences Laboratory, National University of Singapore, Singapore; 4 School of Animal, Rural and Environmental Sciences, Nottingham Trent University, Nottingham, UK; 5 College of Life Sciences, Zhejiang University, Hangzhou, China

**Keywords:** Auxin, gravitropism, OsPIN2, rice, root growth angle, root system architecture

## Abstract

Root system architecture is very important for plant growth and crop yield. It is essential for nutrient and water uptake, anchoring, and mechanical support. Root growth angle (RGA) is a vital constituent of root system architecture and is used as a parameter for variety evaluation in plant breeding. However, little is known about the underlying molecular mechanisms that determine root growth angle in rice (*Oryza sativa*). In this study, a rice mutant *large root angle1* (*lra1*) was isolated and shown to exhibit a large RGA and reduced sensitivity to gravity. Genome resequencing and complementation assays identified *OsPIN2* as the gene responsible for the mutant phenotypes. *OsPIN2* was mainly expressed in roots and the base of shoots, and showed polar localization in the plasma membrane of root epidermal and cortex cells. OsPIN2 was shown to play an important role in mediating root gravitropic responses in rice and was essential for plants to produce normal RGAs. Taken together, our findings suggest that OsPIN2 plays an important role in root gravitropic responses and determining the root system architecture in rice by affecting polar auxin transport in the root tip.

## Teaser

*OsPIN2* is identified as the casual gene responsible for the phenotype of *lta1*, a rice mutant that displays large root angles and a shallow root system architecture, affecting the polar transport of auxin in the root tip.

## Introduction

Roots play a central role in plant growth and development through providing anchorage and taking up nutrients and water from the soil. The root system architecture in the soil determines the scope of resources available to plants and responds to environmental conditions, thus governing the growth and final yields for crop plants (Rogers and Benfey, 2015). Therefore, ideal root system architectures that optimize water and nutrient uptake have been intensively studied in crop breeding and production in recent years. Root growth angle (RGA) is an important parameter of root system architecture in the soil. Large RGAs (shallow root growth) are now being deployed as targets in crop breeding programs for improving nutrient uptake efficiency in stressful soil environments (Lynch, 2013). It has been reported that shallow root systems can promote phosphate uptake from the topsoil in wheat (Manske *et al.*, 2000) and in common bean (Liao *et al.*, 2004; Lynch, 2011). In addition, shallow roots play a vital role in the avoidance of the hypoxic environments and promote the growth of rice (Mano *et al.*, 2005).

Gravitropic response is an important factor that affects RGA (Morita and Tasaka, 2004). Root gravitropism describes the orientation of root growth along the gravity vector. It has been suggested that in Arabidopsis this response requires the coordinated, asymmetric distribution of auxin within the root tip, and depends on the concerted activities of PIN (PIN-FORMED) proteins, AUX1 (AUXIN-INSENSITIVE1), and other members of the auxin transport pathway (Abas *et al.*, 2006). In Arabidopsis, genetic and biochemical studies have revealed the essential roles of the members of PIN, AUX1/LAX (AUXIN-INSENSITIVE1/LIKE AUX1), and ABCB (B subfamily of ABC transporters) families in mediating polar auxin transport and root gravitropism (Geisler *et al.*, 2014). *AUX1* is expressed in root apical tissues, and regulates root gravitropism by facilitating auxin transport in Arabidopsis (Marchant *et al.*, 1999). AXR4 is an accessory protein of the endoplasmic reticulum (ER) and regulates AUX1 localization, thus affecting root gravitropism (Hobbie and Estelle, 1995; Dharmasiri *et al.*, 2006).

There are eight PIN proteins in Arabidopsis (Grunewald and Friml, 2010), among which PIN1 is involved in the basipetal movement of auxin and is crucial for shoot vascular development and shoot gravitropic responses (Gälweiler *et al.*, 1998; Vernoux *et al.*, 2000; Friml, 2003). PIN2 and PIN3 are essential for the root gravitropic response. PIN2 polar localizes towards the shoot in the lateral root cap and root epidermis cells, and towards the root in the root cortex cells. Roots of *pin2* mutant seedlings are agravitropic. In *pin2* mutants, shootward auxin distribution in the lower side of the root is largely repressed during gravity stimulus, thus resulting in an agravitropic phenotype (Chen *et al.*, 1998; Luschnig *et al.*, 1998; Müller *et al.*, 1998; Utsuno *et al.*, 1998). PIN3 localizes in the lower side of the columella cells, and functions in redirecting auxin fluxes to trigger asymmetric growth (Grunewald and Friml, 2010). *pin3* seedlings display decreased root gravitropic responses and inhibited hypocotyl and root growth (Friml *et al.*, 2002; Harrison and Masson, 2008; Keuskamp *et al.*, 2010). While PIN4 and PIN7 have partially overlapping functions with other PINs in the root tip, their involvement in root gravitropism is not clear (Grunewald and Friml, 2010). In addition, the B subclass ABC transporters, such as ABCB1 (PGP1), ABCB19 (PGP19/MDR1), and ABCB4 (PGP4), have been shown to affect root gravitropism since their loss-of-function mutants display significantly reduced gravitropic responses (Noh *et al.*, 2001; Santelia *et al.*, 2005; Terasaka *et al.*, 2005; Bouchard *et al.*, 2006; Bailly *et al.*, 2008)

Rice is a model cereal plant that possesses a fibrous root system, which is mainly composed of crown roots emerging post-embryonically from the nodes of the stem (Coudert *et al.*, 2010). The crown root growth angle is an important component for the distribution of rice roots in soil. Recently, several quantitative trait loci (QTLs) have been identified as contributing to the regulation of RGA in rice (Uga *et al.*, 2013*a*, 2013*b*; Kitomi *et al.*, 2015). One of these, *DEEPER ROOTING 1* (*DRO1*), is negatively regulated by auxin and is also involved in cell elongation in the root tip (Uga *et al.*, 2013*a*). Uga *et al.* (2013*b*, 2015) have also characterized two other major QTLs controlling RGA: one is located on chromosome 4, and the other, *DRO3*, is on the long arm of chromosome 7 and might be involved in the *DRO1* genetic pathway. Although many genes involved in root gravitropic responses have now been identified in Arabidopsis, only a few have been identified in rice. The molecular mechanisms underlying the regulation of RGA and gravitropic responses in rice remain almost unknown.

In this study, a rice root mutant displaying larger root angles and an agravitropic response was isolated, and was named *lra1* (*large root angle1*) according to the phenotype. Molecular cloning and complementation analysis revealed that a point mutation in *OsPIN2* resulted in a premature stop codon and the *lra1* phenotype. Further molecular, genetic, and physiological analyses demonstrated that OsPIN2 plays an important role in rice root gravitropism and in determining the RGA via effects on the polar auxin transport in the root tip.

## Materials and methods

### Plant material and growth conditions

An ethyl-methanesulfonate (EMS)-mutagenized M2 library in the background of *Oryza sativa* L. cv Hei-Jing2 (HJ2), a Japonica rice variety, was used for screening mutants with altered root structure. *lra1*, a mutant with a large root growth angle, was isolated and named according to the phenotype. Rice plants were grown in solution culture (Yoshida *et al.*, 1976) with FeCl_3_ replaced by NaFe(Ⅲ)-EDTA. The pH of the culture solution was adjusted to 5.5 before use and the solution was replaced every 3 d. The solution-cultured mutants and the HJ2 wild-type (WT) were grown in a greenhouse with a 12-h light (30 ℃) / 12-h dark (22 ℃) photoperiod, at approx. 200 μmol m^−2^ s^−1^ photon density, and 60% relative humidity. For an agar-gel medium test, rice seeds were sterilized using 75% ethanol for 2 min and 30% bleach for 30 min with gentle shaking, and then washed for 6∼7 times with sterile double-distilled water. Seeds were dried for 3 min, then laid in half Murashige and Skoog (MS) solid medium in tissue-culture flasks (Thermo Fisher Scientific), and then grown vertically for 7 d before taking photographs.

For imaging with X-ray micro-computed tomography (X-ray μCT), plants were grown in polyvinyl chloride columns (80 mm diameter × 180 mm height) containing sieved (<2 mm) sandy clay loam soil (sand 60%, silt 17%, and clay 23%; pH 7.1; organic matter 5%) (‘Sterilised Kettering Loam’, Broughton Ltd., Kettering, UK). The soil was uniformly packed to a bulk density of 1.2 Mg m^−3^ and saturated overnight from the bottom upwards with deionized water before planting with pre-germinated rice seeds (3 d after germination with emergence of the coleoptile and radicle). Growth conditions were the same as those in the solution culture detailed above.

### Gene cloning and complementation tests

To clone the causal gene, the *lra1* mutant was backcrossed with the HJ2 wild-type. F_2_ progeny plants showing large root growth angles were selected for genetic analysis and gene cloning using the MutMap method (Abe *et al.*, 2012). DNA was extracted from 40 F_2_ individuals and mixed in an equal ratio. Genomic resequencing, single-nucleotide polymorphism (SNP) and InDel analysis, and mutation identification were conducted as previously described (Yang *et al.*, 2016). To confirm that the casual gene *OsPIN2* was mutated in the mutant, the coding sequence and genomic DNA of *OsPIN2* were amplified (primers *OsPIN2*-F, *OsPIN2*-R) from the mutant and HJ2 for sequencing analysis. A cleaved amplified polymorphic sequence (CAPS) marker [primers CAPS-F and CAPS-R (Tsp45I)] was also developed to confirm the mutation of the *lra1* mutant. For complementation tests, the sequence of *OsPIN2* (6245 bp), including the genomic sequence of the *OsPIN2* coding domain (3691 bp) and its native promoter (2554 bp), were PCR-amplified from HJ2 DNA using the primer pairs *OsPIN2*-infusion-F1 (*Eco*RI) and *OsPIN2*-infusion-R1 (*Hind*Ⅲ) and inserted into the binary vector pCambia1300 between *Eco*RI and *Hind*Ⅲ using an Infusion kit (Takara Clontech, Japan). The binary vector was transformed into *lra1* mutants by *Agrobacterium*-mediated transformation (Chen *et al.*, 2003). All the primer sequences are listed in Supplementary Table S1 at *JXB* online.

### β-Glucuronidase (GUS) histochemical analysis

To identify the tissue-specific expression of *OsPIN2*, *pOsPIN2:* GUS transgenic plants were generated. The promoter of *OsPIN2* was amplified using a pair of specific primers: *proOsPIN2*-F (which contains the *Sal* I restriction site) and *proOsPIN2*-R (which contains the *Bam*HI restriction site). The promoter region was cut using *Sal*I and *Bam*HI and cloned into the vector pBI101.3GUSplus (http://genome-www.stanford.edu/vectordb/vector_descrip/COMPLETE/PBI1013.SEQ.html). The resultant *pOsPIN2*:GUS construct was transformed into the WT by *Agrobacterium*-mediated transformation. Histochemical GUS analysis was performed as previously described (Liu *et al.*, 2005). The primers are listed in Supplementary Table S1.

### Antibody preparation, immunostaining, and sub-cellular localization

The OsPIN2 antibodies were prepared and purified as described previously (Li *et al.*, 2014)

For immunostaining, the roots of 1-week-old seedlings of WT and *lra1* were used as described previously (Murata *et al.*, 2006).

The *pOsPIN2:OsPIN2-eGFP* expression vector was constructed as described previously by Wu *et al.* (2015), and the *35S:OsCHL1-mCherry* expression vector was constructed as described previously by Lv *et al.* (2014). The resulting constructs were sequenced to verify the in-frame fusion, and then used for transient transformation in rice protoplasts (Miao and Jiang, 2007). Transformed protoplasts were examined with a confocal microscope (Zeiss LSM 710).

### RNA extraction, reverse transcription, and quantitative RT-PCR

Total RNAs were isolated using an RNA extraction kit (NucleoSpin RNA Plant; Macherey-Nagel, Germany). Reverse transcription, RT-PCR, and quantitative RT-PCR were performed as described by Chen *et al.* (2013). The primers used for RT-PCR and qRT-PCR are listed in Supplementary Table S1.

### Measurements of IAA concentration and distribution

For measurement of indole-3-acetic acid (IAA) concentrations, 20 mg samples of roots of 7-d-old WT and *lra1* mutants were washed several times with deionized water, and ground into fine powder under liquid nitrogen. The measurement of IAA was performed as described previously (Wang *et al.*, 2014). Rice auxin-inducible reporter DR5-GFP lines were obtained by transforming the DR5-GFP reporter gene into both the WT and the *lra1* mutant. The DR5-GFP vector was constructed as described previously (Qi *et al.*, 2012). The auxin distribution revealed by GFP fluorescence in WT and *lra1* roots was visualized using a confocal microscope (Zeiss LSM 710).

### Root gravistimulation and measurement

Root gravistimulation was performed as previously described (Paciorek *et al.*, 2005). For gravitropic stimulation, 5-d-old vertically grown seedlings were gravistimulated with 90° rotation. Digital images were collected for at least 20 seedlings for each time point and analyzed using the Image J software (https://imagej.nih.gov/ij/).

### Identifying the function of *OsPIN2* in Arabidopsis

To understand whether *OsPIN2* functions similarly to *AtPIN2*, a construct of *pAtPIN2*:*OsPIN2* was made as follows: the *AtPIN2* promoter (2836 bp) was amplified by PCR using the primers *proAtPIN2*-infusion-F3 (containing the *EcoRI* restriction site) and *proAtPIN2*-infusion-R3 (containing the *Kpn*I restriction site), and then inserted into the pCAMIBA1300 vector using In-Fusion™ PCR Cloning Kits (Takara Clontech, Japan). Then the cDNA (2119 bp) of *OsPIN2* was amplified by PCR using the primers *OsPIN2*-cDNA-F (containing the *Kpn*I restriction site) and *OsPIN2*-cDNA-R (containing the *Kpn*I restriction site) and inserted into the pCAMIBA1300 vector containing the promoter of *AtPIN2* at the *Kpn*I site using In-Fusion™ PCR Cloning Kits (Takara Clontech, Japan). The resulting *pAtPIN2*:*OsPIN2* construct was transformed to *Atpin2* mutants to get the *pAtPIN2*:*OsPIN2*/*Atpin2* transgenic lines using the floral dip method (Zhang *et al.*, 2006). All the primers are listed in Supplementary Table S1.

### 3D reconstruction of root structure based on X-ray µCT scanning

X-ray μCT scans were performed at The Hounsfield Facility (School of Biosciences, The University of Nottingham, UK) using a phoenix v|tome|x m 240kV X-ray CT system (GE Sensing & Inspection Technologies GmbH, Wunstorf, Germany). Two scans were required per plant to obtain the full column height (which were digitally combined following data reconstruction). Each scan acquired 2160 projection images over a 360° rotation of the sample using a detector exposure time of 250 ms, integrated over three averaged images, resulting in a total scan time of 75 min for both scans. Full details of the X-ray CT scanner settings are given in Supplementary Table S2. Following the scans, data were reconstructed using the datos|REC software (GE Sensing & Inspection Technologies GmbH, Wunstorf, Germany). Visualization and quantification of root material in the reconstructed data were preformed using the method of Tracy *et al.* (2012), employing a combination of the VGStudioMAX v2.2 (Volume Graphics GmbH, Heidelberg, Germany) and RooTrak software (Mairhofer *et al.*, 2012). For the measurement of root angles, a bespoke software tool called RooTh was used (Mairhofer, *et al.*, 2017).

## Results

### Isolation and phenotypic characterization of the *lra1* mutant

**Fig 1.**
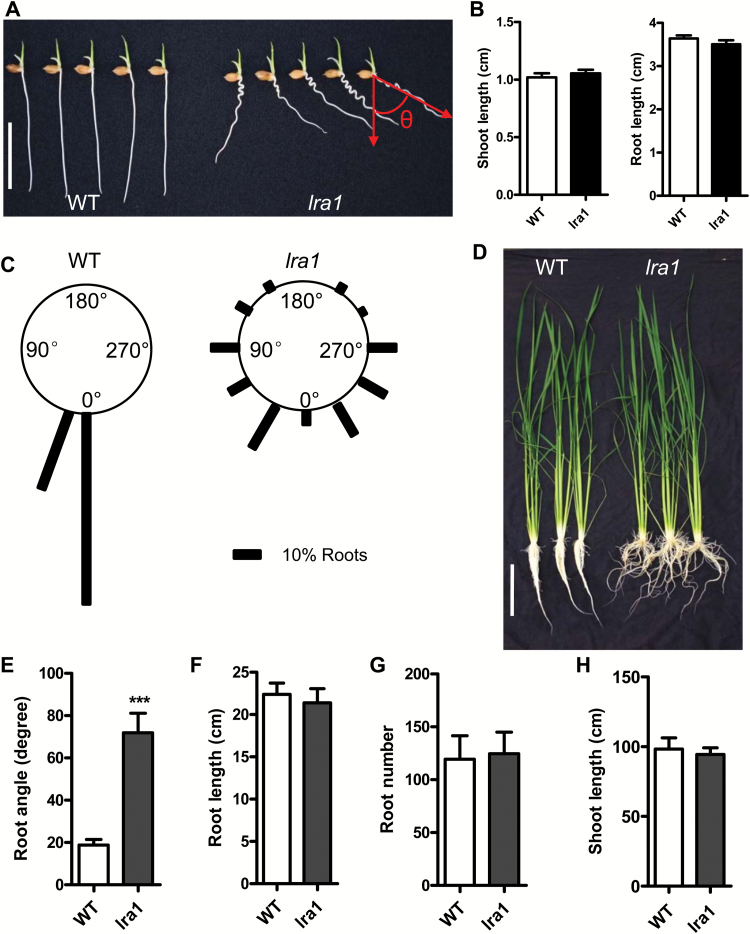
Phenotype of the *lra1* mutant and the wild-type (WT). (A) 3-d-old WT and *lra1* seedlings grown in solution culture. Scale bar =2 cm. θ indicates the root angle. (B) Shoot and root lengths of 3-d-old WT and *lra1* seedlings as shown in (A). Data are means ±SE (*n*=20). (C) The root gravitropic response of 3-d-old WT and *lra1* (*n*=100). (D) Phenotype of 4-week-old WT and *lra1* seedlings grown in solution culture. Scale bar=15 cm. (E–H) Root angle (E), root length (F), root number (G), and shoot length (H) of 4-week-old WT and *lra1* seedlings grown in solution culture. Data are means ±SD (*n*=10). Asterisks indicate significance differences between WT and *lra1* plants as determined by Student’s *t*-test (***, *P*<0.005).

To investigate the molecular mechanisms governing the root system architecture in rice, we obtained a rice root mutant *lra1* in a screening of an EMS library. Compared with the WT, 3-d-old *lra1* mutant seedlings displayed an agravitropic phenotype with slightly curved roots (Fig. 1A). However, the root and shoot lengths of the seedlings showed no significant differences between *lra1* and the WT (Fig. 1B). Root angles of WT seedlings were mainly between 0–20°, but those of *lra1* seedlings varied from 0 to 360° (Fig. 1C).

The primary root length, shoot length, root number, lateral root number, longest lateral root length, and the total lateral root length were not significantly affected in the *lra1* mutant compared with the WT after 7 d in solution culture (Supplementary Fig. S1), but *lra1* showed larger root growth angle (Supplementary Fig. S2A) and a reduced gravitropic response (Fig. S2B). After 4 weeks, the average root angle of *lra1* mutants (71.9°) was much larger than that of the WT (18.75°) (Fig. 1E, Supplementary Fig. S2C). These results indicated that both the primary root and the later-emerging adventitious roots in *lra1* showed reduced gravitropic responses (Supplementary Fig. S2A–C). There were no significant differences in shoot length, root length, and root number in 4-week-old seedlings grown in solution culture between *lra1* and WT (Fig.1F–H).

At the reproductive stage in solution culture, the *lra1* mutant showed no significant differences in agronomic traits such as plant height, tiller number, and seed-setting compared with the WT (Supplementary Table S3). However, the plant height of *lra1* was significant lower than that of the WT, although the other measured traits showed no difference with the WT in soil culture (Supplementary Fig. S3 and Supplementary Table S4).

### Cloning and identification of *LRA1*

**Fig 2.**
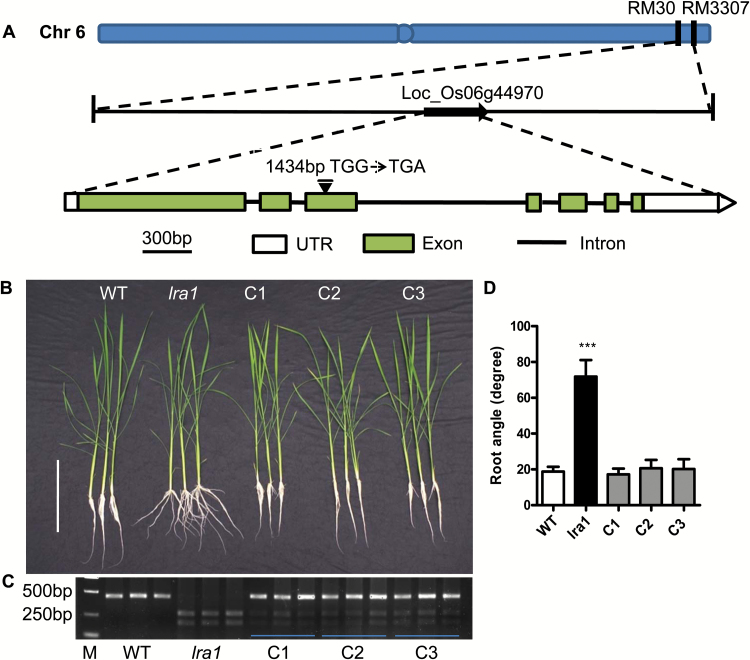
Gene cloning and complementation analysis. (A) Chromosome location and gene structure of *OsPIN2*. The G-to-A mutation on the third exon is indicated. (B–D) Complementation analysis. (B) Phenotype of 30-d-old seedlings of the wild-type (cv HJ2), *lra1* mutant, and three complementation (*OsPIN2p:OsPIN2/lra1*) lines (C1–C3) grown in hydroponics. Scale bar =20 cm. (C) Molecular characterization of the complementation lines by CAPS maker (primers are listed in Supplementary Table S1, PCR products were digested with *Tsp45*I). M, molecular marker (DL2000, Takara). (D) The root angle of seedlings shown in (C). Data are means ±SD of ten replicates. Asterisks indicate significance differences as determined by Student’s *t*-test (***, *P*<0.005).

To identify the casual gene of the *lra1* mutant, we backcrossed the mutant to the WT. All the plants of the F_1_ progeny showed the small root angle of the WT. In the F_2_ progeny, 172 and 59 plants showed the WT and mutant phenotypes, respectively, which fits a ratio of about 3:1 (χ^2^=9.9, *P*<0.01). This result indicates that the *lra1* mutant is caused by a single recessive gene. After genome resequencing of the bulked mutant-like plants (*n*=39) and WT plants (*n*=30) in the F_2_ progenies, a single nucleotide mutation (G1434A) was found in the third exon of LOC_Os06g44970, which generated a premature stop codon (Fig. 2A). The point mutation in the *lra1* mutant was confirmed using a CAPS marker (Fig. 2C). LOC_Os06g44970 is annotated as an auxin efflux transporter OsPIN2 that contains nine transmembrane domains. The mutation in the *lra1* mutant would produce a truncated OsPIN2 that has lost the last four transmembrane segments, as predicted by InterPro (http://www.ebi.ac.uk/interpro/search/sequence-search;Supplementary Fig. S4).

In order to confirm the fact that the agravitropic phenotype of *lra1* was caused by the point mutation of *OsPIN2*, the genomic sequence of *OsPIN2* driven by its native promoter was transformed to the *lra1* mutants. A series of transgenic lines were obtained that showed the same small root angles as the WT. Three representative lines were molecularly characterized using a CAPS marker and showed the bands of *lra1* and band of the WT, indicating their transgenes in the *lra1* background (Fig. 2). Phenotypic analysis was conducted using the three lines. The root angles of the complementary transgenic lines were comparable to those of WT plants in different growth media (Fig. 2B, D; Supplementary Fig. S2A–C), and the expression levels of *OsPIN2* in the complementary lines were comparable with those in the WT (Supplementary Fig. S2D). The results suggest that the phenotypic defect of *lra1* is caused by the mutation of *OsPIN2*.

### Expression pattern of *OsPIN2*

**Fig 3.**
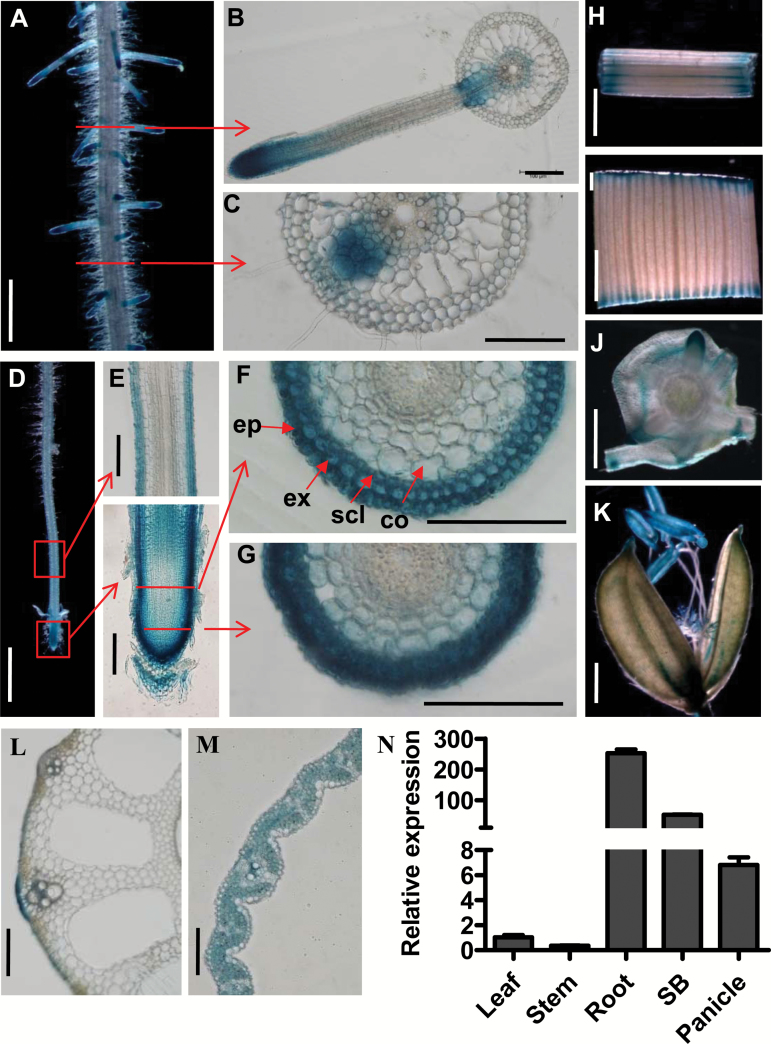
Tissue-specific expression patterns of *OsPIN2*. (A–M) GUS staining of transgenic plants harboring the *OsPIN2p:GUS* reporter gene. (A) Mature root region with lateral roots, (B) cross-section of the region where a lateral root emerges, (C) cross-section of the region where a lateral root primordium is initiated, (D) primary root tip, (E) longitudinal section of primary root tip (lower panel) and the elongation zone (upper panel), (F) cross-section of the elongation zone, (G) cross-sections of a root cap, (H) stem, (I) leaf, (J) stem base, and (K) panicle. Abbreviations: ep, epidermis; ex, exodermis; scl, sclerenchyma; co, cortex. Scale bars indicate 1 mm in (A, D, H–K), 100 µm in (B, C, E–G, M), 200 µm in (L). (N) Quantitative RT-PCR analysis of *OsPIN2* mRNA levels in the leaf, stem, root, stem base (SB), and panicle. The primers used are listed in Supplementary Table S1.

The tissue-specific expression pattern of *OsPIN2* was analysed by using transgenic lines in which the GUS reporter gene was driven by the *OsPIN2* promoter in WT plants. GUS staining of the representative transgenic lines revealed that *OsPIN2* was expressed in lateral root tips (Fig. 3A, B), primary root tips (Fig. 3D), stems (Fig. 3H), leaves (Fig. 3I), the stem base (Fig. 3J), and flowers (Fig. 3K). Cross-sections of different parts of a primary root showed that *OsPIN2* was expressed in lateral root primordia and lateral root initiation zones (Fig. 3B, C). Longitudinal and cross-sections of primary root tips showed that *OsPIN2* was mainly expressed in the epidermis, exodermis, and sclerenchyma cells of the root meristematic and elongation zones (Fig. 3E–G). Cross-sections of stems and leaves demonstrated that *OsPIN2* was also expressed in the epidermal tissues of the stem (Fig. 3L) and in leaf mesophyll cells (Fig. 3M). The results were in agreement with the expression patterns of *OsPIN2* that have been described previously (Wang *et al.*, 2009; Chen *et al.*, 2012).

Quantitative RT-PCR analysis showed that the expression levels of *OsPIN2* were much higher in roots and the stem base (SB) than in leaves, stems, and panicles (Fig. 3N), implying that OsPIN2 plays a role in root development in rice.

### Cellular and subcellular localization of OsPIN2

**Fig 4.**
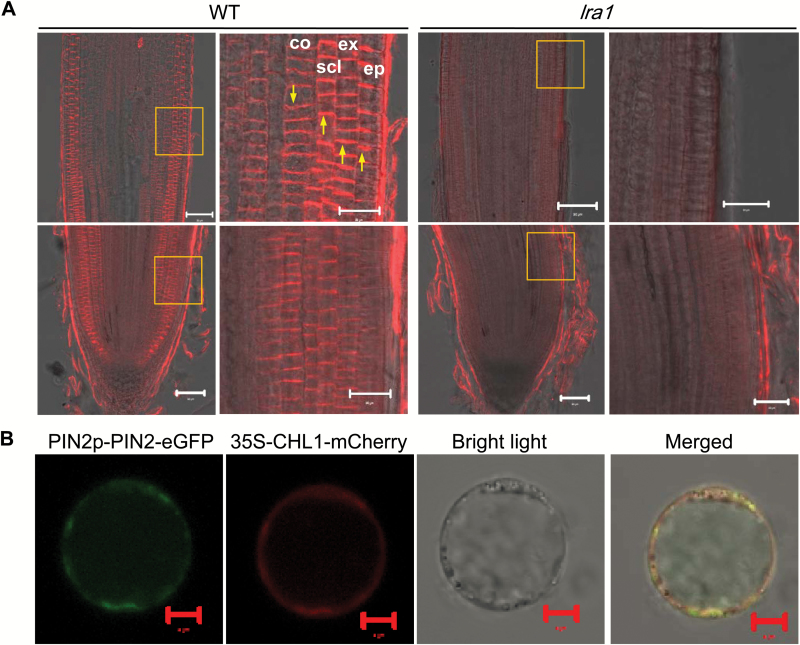
Tissue-specificity and subcellular localization of OsPIN2. (A) Tissue-specificity in the root tip. Immunostaining was performed with an anti-PIN2 antibody in root tips of the wild-type (WT) and *lra1* (longitudinal sections). The boxed areas are magnified in the images to the right. Abbreviations: ep, epidermis; ex, exodermis; scl, sclerenchyma; co, cortex. Arrows indicate polar localization of OsPIN2. Scale bars =20 µm. (B) Subcellular localization of OsPIN2. The OsPIN2-GFP fusion protein driven by the *OsPIN2* promoter and CHL1-mCherry (cell membrane marker) driven by the 35S promoter were transiently co-expressed in rice protoplasts. Fluorescent signals as observed by confocal microscopy are shown. Scale bars =5 µm.

The cellular localization of the OsPIN2 protein was examined by immunostaining using an anti-OsPIN2 polyclonal antibody. In WT plants, signals were observed on the plasma membranes of epidermal and cortex cells in the root tip, and polar localization could be seen on the upper side in epidermal cells and on the lower side in cortex cells (Fig. 4A). In general, the signals in epidermal cells were much stronger than those in cortex cells (Fig. 4A). In contrast, no signals were detected in *lra1* roots. These observations confirm that the OsPIN2 protein is truncated in *lra1* mutants.

The subcellular localization of OsPIN2 was further examined by transient expression in rice protoplasts. The OsPIN2 protein was co-localized with CHL1, a plasma-membrane marker (Lv *et al.*, 2014) (Fig. 4B), indicating that OsPIN2 was indeed localized in the plasma membrane.

### *OsPIN2* rescues the phenotypic defect of the *pin2* mutant in Arabidopsis

**Fig 5.**
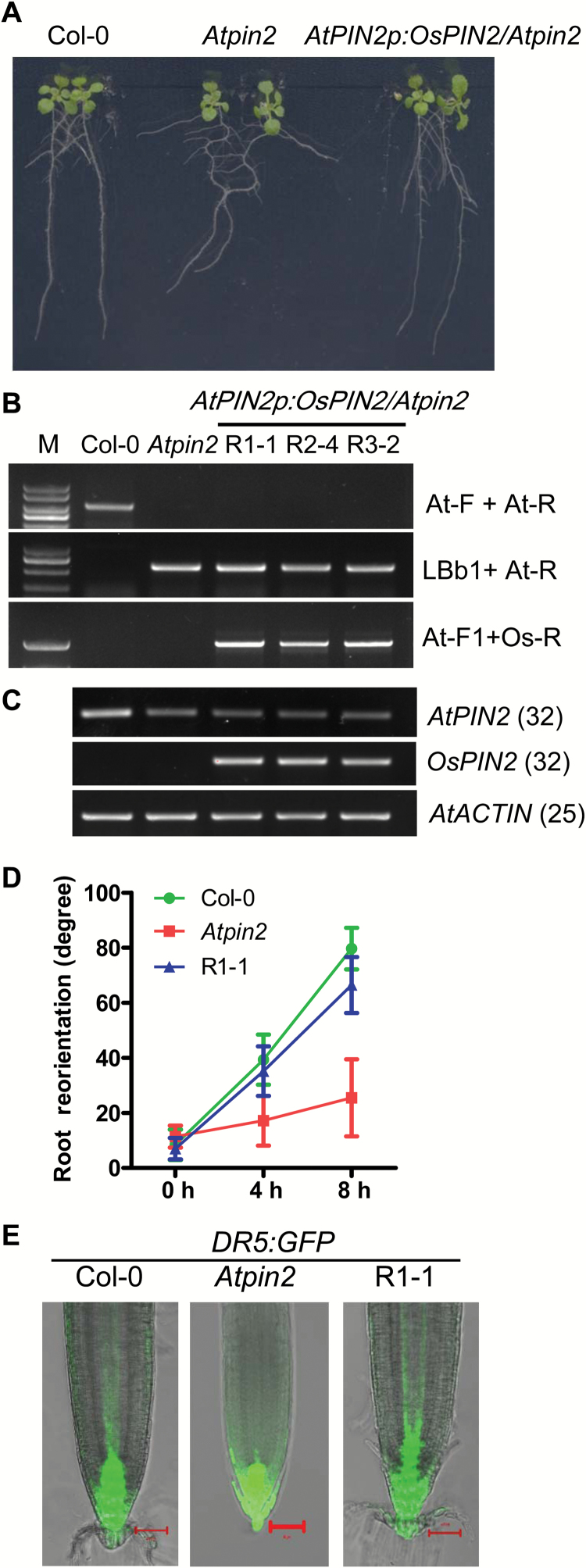
Expression of *OsPIN2* rescues the phenotype of the Arabidopsis *pin2* mutant. (A) The phenotypes of 10-d-old Col-0, *Atpin2*, and *Atpin2p:OsPIN2/Atpin2* transgenic lines. (B) Molecular characterization of Col-0, *Atpin2*, and three independent *Atpin2p:OsPIN2/Atpin2* transgenic lines using PCR. Primers are listed in Supplementary Table S1. (C) Expression levels of *AtPIN2* and *OsPIN2* in the transgenic lines using RT-PCR. (D) Kinetics of root reorientation of Col-0, *Atpin2*, and *Atpin2p:OsPIN2/Atpin2* transgenic lines (R1-1): 4-day-old seedlings were placed horizontally and the root angle was measured at time points as indicated. Data are means ±SD (*n*=20). (E) Fluorescence of *DR5:GFP* in Col-0, *Atpin2*, and *Atpin2p:OsPIN2/Atpin2* line R1-1; scale bars =50 μm.

To test whether OsPIN2 functions like AtPIN2 as an auxin efflux carrier (Müller *et al.*, 1998), the *OsPIN2* full-length cDNA driven by the *AtPIN2* promoter was transformed into the Arabidopsis *pin2* mutant (*Atpin2*). The transgenic lines, which were verified by PCR (Fig. 5B, C), showed normal root growth just like the wild-type plants (Fig. 5A). Gravitropic root reorientation was examined to test whether the response of the transgenic seedlings was rescued. The kinetics of root reorientation of the WT, *Atpin2*, and the transgenic line (R1-1) were examined when roots were placed horizontally for up to 8 h. The transgenic line showed normal root reorientation similar to that of the WT, while the *Atpin2* mutant displayed an agravitropic response (i.e. as seen in Fig. 5A). Furthermore, the root reorientation of R1-1 was comparable to that of the WT, while *Atpin2* did not respond to gravistimulation (Fig. 5D). These results indicated that the gravitropic response of *Atpin2* was recovered to the level of the WT by expressing *OsPIN2* (Fig. 5A). To test whether the auxin distribution in the root tips of R1-1 was rescued, lines of *DR5-GFP*/*Atpin2* and *DR5-GFP*/R1-1 were constructed. In contrast to *Atpin2*, the auxin distribution as revealed by the GFP signal in the R1-1 line was the same as that in the WT, and was mostly located in the columella, stele, and epidermis (Fig. 5E). This indicates that OsPIN2 can rescue the defective auxin distribution in *Atpin2*. These results suggest that OsPIN2 plays a similar role to Arabidopsis PIN2 as an auxin efflux carrier that regulates the root gravitropic response in rice.

### The distribution of auxin is altered in root tips of *lra1* mutants

**Fig 6.**
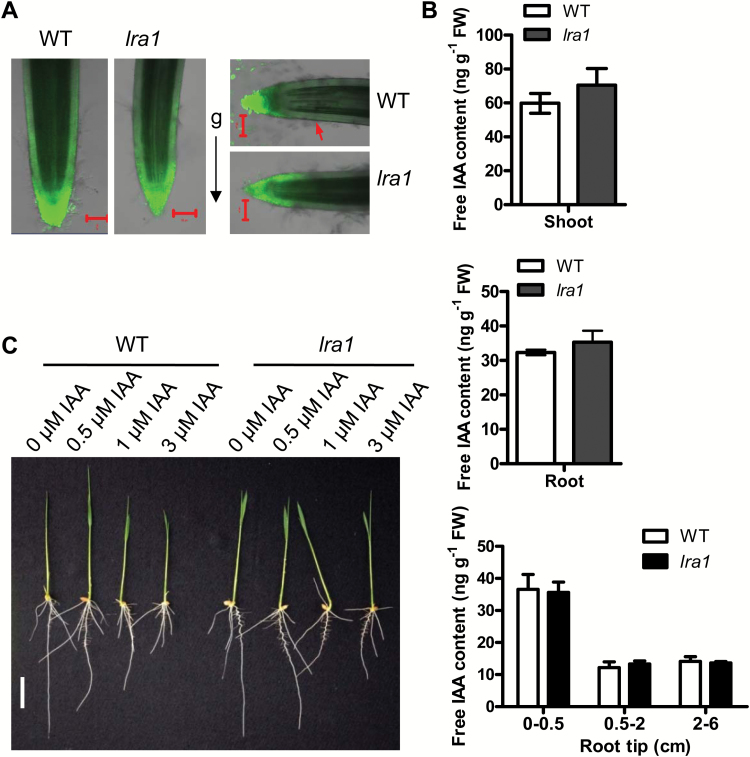
Mutation of OsPIN2 affects the distribution of auxin in rice root tips. (A) Auxin distribution as revealed by green fluorescence in the roots of 3-d-old *DR5:GFP* transgenic wild-type (WT) and *lra1* plants. The roots shown in the panels to the right were placed horizontally for 30 min before imaging (g indicates direction of gravity). Images are representative of three independent lines. (B) Endogenous free IAA concentrations in different tissues of 7-d-old WT and *lra1* mutant plants. Data are means ±SE of three replicates. (C) Phenotypes of WT and *lra1* seedlings treated with different concentrations of IAA, as indicated.

To test whether the distribution of auxin in root tips was different between WT and *lra1* seedlings, the DR5-GFP reporter line was developed and crossed with the *lra1* mutant. As revealed by GFP fluorescence, the auxin signal in root-cap columella cells of the *lra1* mutant was less than that in the WT; no significant differences were observed in the other tissues in the root tip (Fig. 6A). After being placed horizontally for 30 min, GFP fluorescence was higher on the lower side of the root cap in the WT, but not in the *lra1* mutant (Fig. 6A). This indicates that *OsPIN2* plays an important role in auxin polar distribution in the root tips.

The concentrations of endogenous free IAA in the WT and *lra1* seedlings were further quantified. As shown in Fig. 6B, there were no differences in concentrations at the whole-shoot and whole-root level between the WT and *lra1*. Further analysis showed that there were also no differences when individual sections of the roots were examined at various differences from the root tip.

To examine whether the external IAA would affect the phenotype, different concentrations of IAA were applied to WT and *lra1* plants. The results showed that *lra1* plants were less sensitive to external IAA compared with WT plants, especially at a concentration of 0.5 μM IAA (Fig. 6C, Supplementary Fig S5).

### OsPIN2 plays an important role in the root system architecture

**Fig 7.**
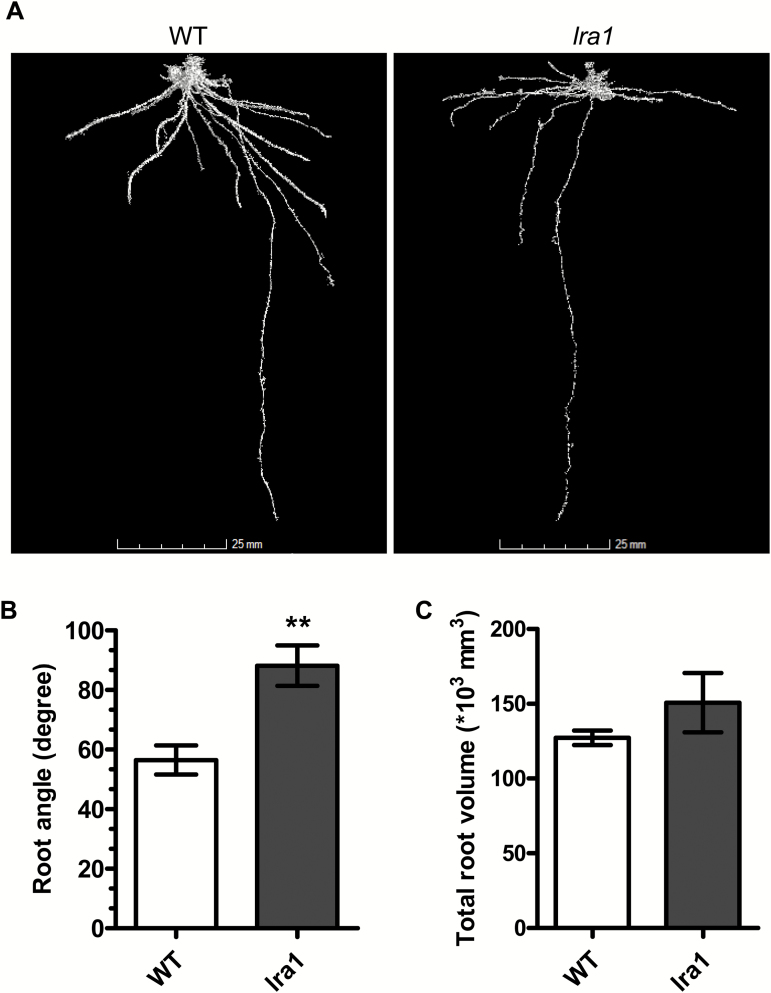
Root system architecture of wild-type (WT) and *lra1* plants as revealed by X-ray computed tomography (CT). (A) Root system architecture of the WT (cv HJ2) and *lra1* grown for 21 d in 8-cm diameter pots filled with sterilized Kettering loam. Roots were segmented by a region-growing algorithm. (B) Root angles of the WT and *lra1* measured using the software RooTh (provided by University of Nottingham). Asterisks indicate significance differences as determined by Student’s *t*-test (**P<0.01). (C) Total root volume of the WT and *lra1* measured using the software Rootrak_0.3.2 (provided by University of Nottingham).

X-ray computed tomography (μCT) is a non-destructive imaging method that permits 3D reconstruction of scanned objects (Tracy *et al.*, 2010). To determine whether *lra1* affected the root architecture in of plants grown in soil, 3D root systems of *lra1* and WT plants were reconstructed using μCT imaging (representative images are shown in Fig. 7A, and additional images from different angles are also presented in Supplementary Fig. S6). The images revealed that roots of *lra1* plants had a larger root angle (88.1°) and were distributed more in the upper surface layer of the soil compared to the WT plants (56.4° and deeper root growth) (Fig. 7A, B), which is consistent with what was observed in solution culture and in MS medium (Supplementary Fig. S2). The total root volume showed no significant difference between the WT and *lra1* (Fig 7C). These results suggest that OsPIN2 plays an important role in root system architecture by affecting the root growth angle.

SNPs within *OsPIN2* among different rice varieties were analysed using RiceVarMap (http://ricevarmap.ncpgr.cn/). The results showed that there are 21 SNPs within *OsPIN2* among 799 Indica varieties and 497 Japonica varieties. Of the 21 SNPs, four were synonymous, 15 were localized in the intron or in the 3′ untranscribed region (3′UTR) (Supplementary Table S5), and two non-synonymous SNPs (sf0627201163 and sf0627201316) showed differentiation between Indica and Japonica. sf0627201163 displayed A in 98.25% of Indica rice and G in 99.2% of Japonica, while sf0627201316 displayed G in 98.12% of Indica rice and T in 99.2% of Japonica (Supplementary Table S5).

## Discussion

Root gravitropism is a complex process during which plant roots grow downwards into the soil. The gravitropic response process contains four steps: sensing the direction of gravity; conversion of a biophysical signal to a biochemical one; transmission of the signal to the responding tissues; and organ bending (Morita and Tasaka, 2004; Philosoph-Hadas *et al.*, 2005). Although the genes involved in root gravitropic responses in Arabidopsis have been well studied, how the response is regulated in rice is still largely unknown. Using forward genetic analysis, this study has identified *LRA1* in rice, a gene encoding the auxin efflux transporter OsPIN2, which is required for the root gravitropic response. A single-nucleotide mutation (G1434A) in *lra1* produces a truncated OsPIN2 protein without the last four transmembrane segments (Supplementary Fig. S4). The *lra1* mutant showed an agravitropic root phenotype with large root growth angles, which could be rescued by the *OsPIN2* genomic sequence (Fig. 2B; Supplementary Fig. S2A–D). After roots were placed horizontally, GFP fluorescence in *DR5:GFP* reporter lines was higher in the lower side of the root tip in the WT, but not in the *lra1* mutant (Fig. 6A). Expression of *OsPIN2* driven by the promoter of *AtPIN2* was able to fully rescue the phenotypic defect in the *Atpin2* mutant (Fig. 5). These results suggest that OsPIN2 plays an important role in root gravitropic responses and in the root growth angle in rice.

### OsPIN2 affects auxin polar distribution in the root tip

Polar auxin transport and redistribution are essential for root gravitropism (Abas *et al.*, 2006). In Arabidopsis, PIN2 (also named AGR1/EIR1/WAV6) localizes towards the shoot in the lateral root cap and root epidermis cells, and towards the root in the root cortex cells, and is known to be an auxin efflux carrier that facilitates basipetal transport (Chen *et al.*, 1998; Luschnig *et al.*, 1998; Müller *et al.*, 1998; Utsuno *et al.*, 1998). Although PIN2 has been shown to mediate basipetal transport of auxin and gravitropic root bending in Arabidopsis (Feraru and Friml, 2008), it is mostly unknown whether PIN2 orthologs play similar roles in other plants.

Our results showed that OsPIN2 localizes on the plasma membrane of epidermal and cortex cells in the root tip (Fig. 4A). After reorientation of roots to change the direction of the gravity stimulation, auxin redistribution was not changed in the *lra1* mutant, as determined by *DR5:GFP* reporter lines (Fig. 6A). Furthermore, *lra1* was less sensitive to external auxin (IAA) treatment compared with WT plants (Fig. 6C, Supplementary Fig. S5). These results suggest that, like PIN2 in Arabidopsis, OsPIN2 plays an important role in polar auxin distribution in root tips.

There are 12 PIN family members in the rice genome (Wang *et al.*, 2009). To investigate whether loss of function of PIN2 affected the expression of other PIN genes, the expression of these genes (*OsPIN1a*, *OsPIN1b*, *OsPIN1c*, *OsPIN3a*, *OsPIN3b*, *OsPIN4*, *OsPIN5a*, *OsPIN5b*, *OsPIN9*, *OsPIN10a*, and *OsPIN10b*) were evaluated in *lra1* by qRT-PCR. The results indicated that the transcript levels of *OsPIN3b* and *OsPIN10b* in shoots were significantly up-regulated in *lra1* compared with the WT. In roots, *OsPIN3a* showed a notable up-regulation in *lra1* compared with that in the WT (Supplementary Fig. S7). Phylogenetic analysis has suggested that *OsPIN3a* and *OsPIN3b* are much more closely related to *AtPIN3* compared with other rice PINs (Miyashita *et al.*, 2010). AtPIN3, mainly located in the columella cell boundaries, is essential for the root gravitropic response (Miyashita *et al.*, 2010). This suggests that loss of function of OsPIN2 up-regulates the expression of OsPIN3, which to some extent compensates for the loss of OsPIN2 function. This is consistent with reports in Arabidopsis that different PINs are ectopically expressed in *pin* mutants and thus can at least partially compensate for the function of the missing PIN protein (Blilou *et al.*, 2005). It is interesting that the transcript levels of the *OsPIN2* were greatly decreased in the *lra1* mutant both in the shoot and root compared with the WT (Supplementary Fig. S7). Whether functional PIN2 is required for the stability of its transcript level in rice or the G-to-A replacement causes instability of the PIN2 mRNA remains to be investigated.

### PIN2 shows conserved function in root gravitropic responses between monocots and dicots

Phylogenetic analysis indicates that OsPIN2 is the closest rice homolog of Arabidopsis PIN2. The rice PIN2 mutant *lra1* showed an agravitropic root phenotype with no significant auxin redistribution to the lower root epidermis cells after roots were reorientated to the horizontal, as determined by *DR5:GFP* (Fig. 6A). These results are consistent with those found in the Arabidopsis *pin2* mutant. AtPIN2 is an auxin efflux transporter (Chen *et al.*, 1998; Luschnig *et al.*, 1998). To understand whether OsPIN2 functions the same as AtPIN2, we transformed a construct of *OsPIN2* driven by the promoter of *AtPIN2* to the *Atpin2* mutant. The transgenic lines showed normal growth and root gravitropic responses just like WT plants (Fig. 5A). The *DR5:GFP* reporter lines also indicated that the defect of auxin distribution in the root tip was also completely rescued by the expression of OsPIN2 (Fig. 5E). These results suggest that the function of OsPIN2 is conserved in root gravitropic responses between monocots and dicots. They also suggest that OsPIN2 might be an auxin efflux transporter in rice. However, differences exist between rice and Arabidopsis. In rice, the fluorescence in the *DR5:GFP* lines was weaker in the root cap columella cells of the *lra1* mutant than in the WT (Fig. 6A). Although the IAA content in the root tip showed no significant differences between *lra1* and the WT (Fig. 6B), this could be because our sampling region encompassed 0.5 cm of the root tip, which not only contained the root cap but also the meristematic region. On the other hand, whether the *DR5:GFP* lines can indicate the auxin content in rice still needs to be verified. In Arabidopsis, *DR5:GFP* lines showed comparable GFP florescence in columella cells between the WT and *pin2* (Fig. 5E). Furthermore, it has been reported that the *Atpin2* mutant responds normally to externally applied auxin (Luschnig *et al.*, 1998), while the *lra1* mutant showed less sensitivity to external auxin, especially at a concentration of 0.5 μM IAA (Fig. 6C, Supplementary Fig S5). The results suggest that *OsPIN2* and *AtPIN2* may affect auxin distribution in different ways, and this needs to be investigated further.

### OsPIN2 is a potential candidate for improving root structure

Optimization of root system architecture is an important objective for modern plant breeding, and hence a better understanding of the molecular mechanisms controlling root architecture is essential to improve plant root systems to enhance nutrient uptake efficiency and crop yield. It has been suggested that root systems with large root angles enhance topsoil foraging and that this benefits the acquisition of phosphate in the upper soil layers (Lynch, 2013). Rice is usually grown in lowland conditions and, in most cases, fields are flooded. Fertilizer use efficiency is a very important parameter for sustainable rice production, and the aim is to reduce the amount of fertilizer applied while keeping yields high. As most nutrients are applied in the surface of the soil, a shallow root system should benefit uptake (Pothuluri *et al.*, 1986; Murphy *et al.*, 1998; Lynch and Brown, 2001; Zhu *et al.*, 2005). It has also been reported that shallow root systems play an important role in the avoidance of hypoxic environments and promote the growth of rice (Mano *et al.*, 2005). *lra1* showed root growth similar to that observed in shallow-rooted varieties, with normal shoot growth irrespective of whether it was cultured in solution or in soil (Figs 1 and 7), which indicates that OsPIN2 plays an important role in root structure. In addition, among the 21 SNPs that were found in different rice varieties, two non-synonymous ones showed different allele priorities between Indica and Japonica varieties. The sf0627201163 SNP showed A in 98.25% of Indica rice and G in 99.2% Japonica, while sf0627201316 showed G in 98.12% of Indica rice and T in 99.2% of Japonica (Supplementary Table S5). This suggests that these two SNPs could be useful for breeding purposes. Overall, our results suggest that *OsPIN2* is a potential candidate gene for improving root system architecture in rice, and possibly other crops.

## Supplementary data

Supplementary data are available at *JXB* online.

Fig. S1. The phenotype of the *lra1* mutant.

Fig. S2. The root phenotype of the wild-type, *lra1* mutant, and complementation lines in different growth media.

Fig. S3. The phenotype of the wild-type and *lra1* mutant grown in soil pots.

Fig. S4. Predicted transmembrane topology models of the OsPIN2 protein in the wild-type and *lra1* mutant.

Fig. S5. Phenotypic data for wild-type and *lra1* seedlings treated with different concentrations of IAA.

Fig. S6. 3D visualization of rice roots grown in soil and viewed from different angles at 21 d after germination.

Fig. S7. qRT-PCR analysis of the expression of PIN family genes in shoots and roots of the wild-type and *lra1* mutant.

Table. S1. Primers used in the study.

Table. S2. Sample properties and scanning settings for X-ray micro-computed tomography.

Table. S3. Agronomic traits of the wild-type and *lra1* grown in solution culture.

Table. S4. Agronomic traits of the wild-type and *lra1* grown in soil pots.

Table. S5. SNPs within *OsPIN2* in different rice varieties.

Supplementary Figures and Table

## Author contributions

MC, WL, PJ, and LC planned and designed the research; WL, GM. and LY performed the experiments; RW, MX, and WZ analysed the data; WL, MC, YH, CJS, and LC wrote the manuscript.
