# Supplementary material for: LARGE ROOT ANGLE1, encoding OsPIN2, is involved in root system architecture in rice
Source: J Exp Bot. 2017 Dec 22;69(3):385–97. doi: 10.1093/jxb/erx427 (PMC5853395; doi:10.1093/jxb/erx427)
Supplement: Supplementary Figures and Table [file erx427_suppl_supplementary_figures-and-table.pdf]

Figure S1

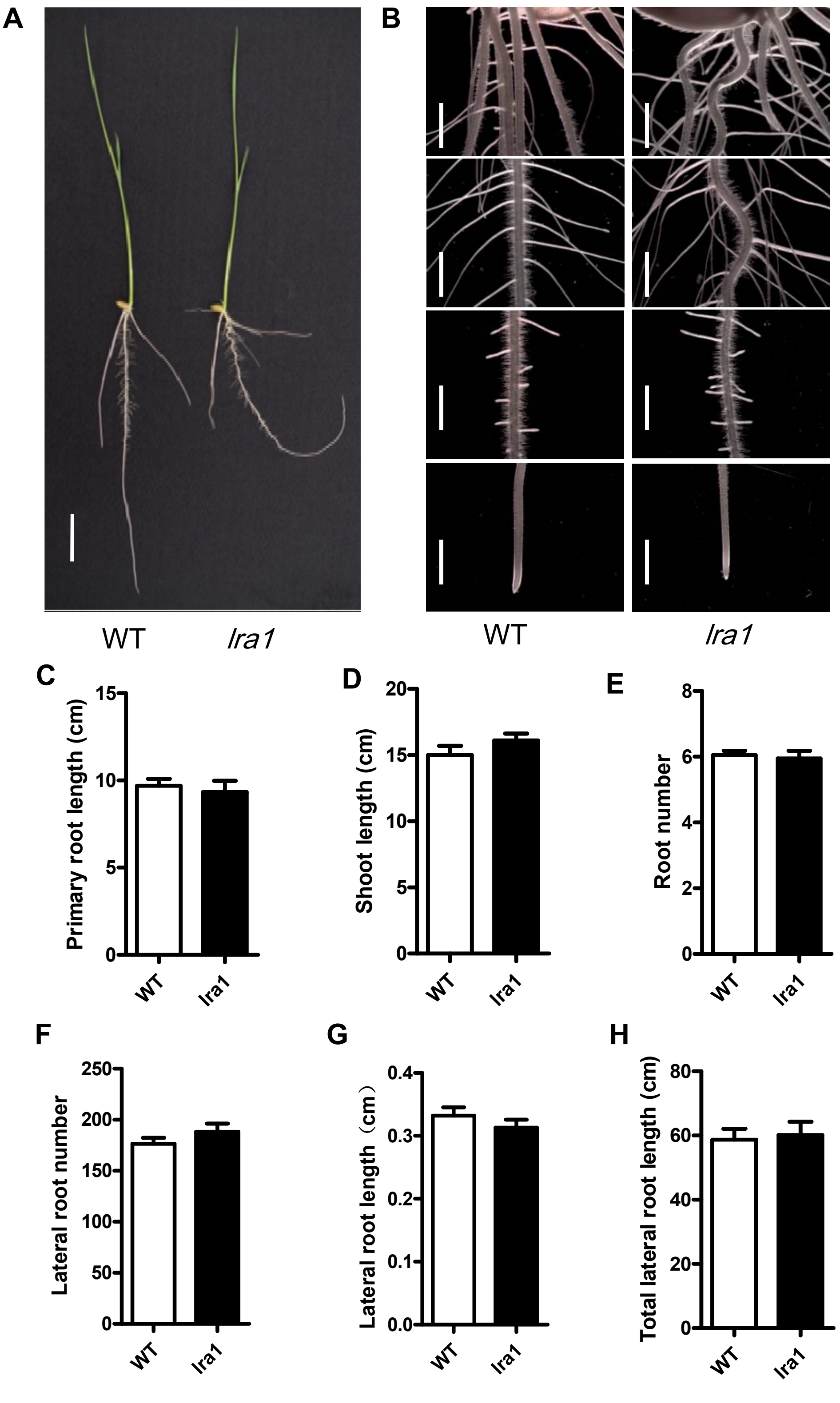

Figure S1. Phenotype of *lra1* mutant.

(A) Phenotype of 7-d-old wild-type (WT) and *lra1* grown in rice culture solution. Bar = 2 cm.

(B) Stereomicroscope images of roots of 7-d-old WT(left) and *lra1*(right) grown in rice culture solution. Scale bar = 1 mm. (C-H) The root traits of 7-d-old WT and *lra1* seedlings grown in rice culture solution. Data are mean ± SE (n=20).

Figure S2

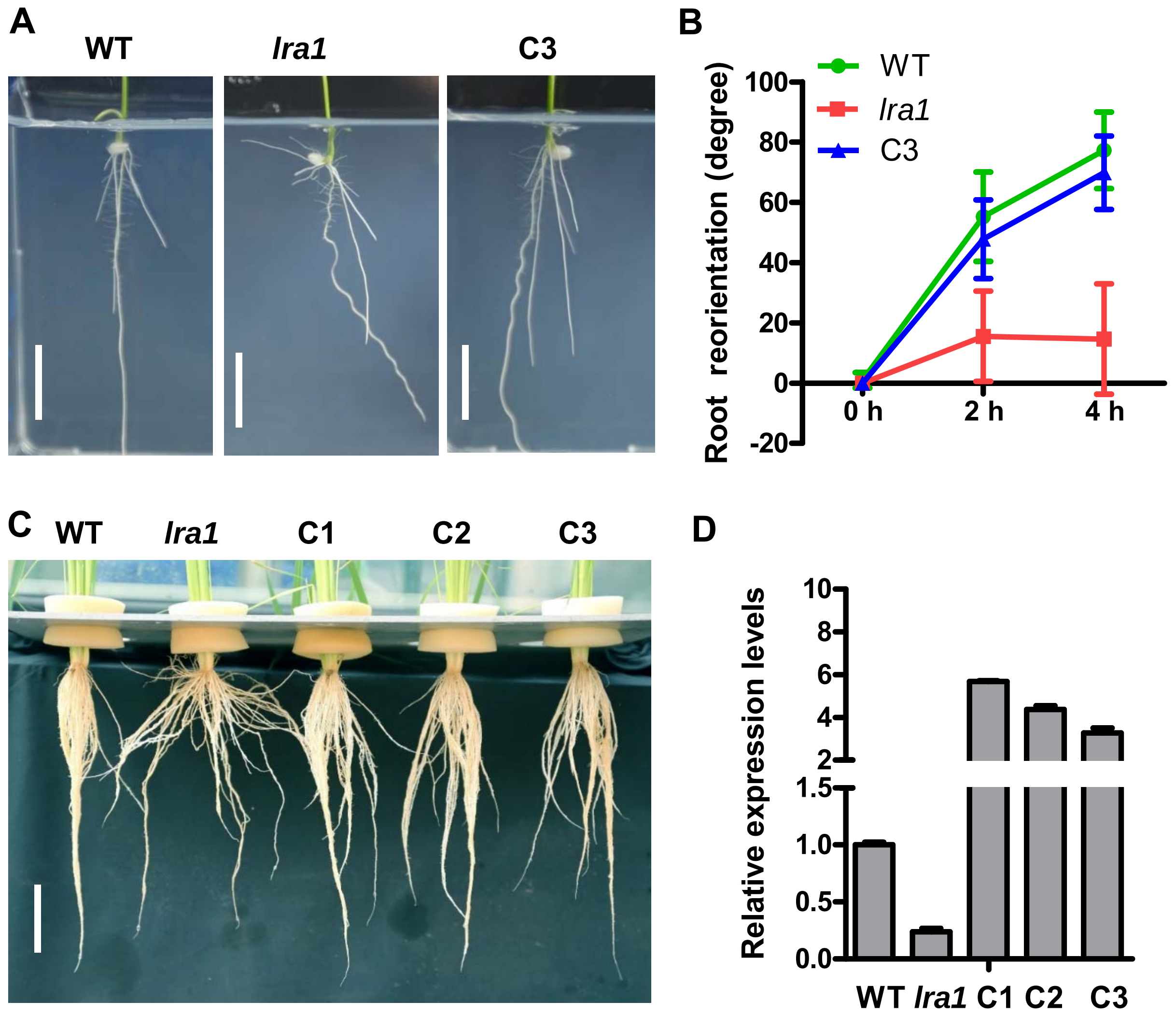

Figure S2 Root phenotype of wild type (WT), *lra1* mutant and complementation lines (C1-C3) in different growth medium. (A) Root phenotype of 7-d-old seedlings grown in half Murashige and Skoog (MS) solid medium. Scale bar = 2 cm. (B) Kinetics of root reorientation of 5-day-old seedlings. Seedlings were horizontally placed, and the root angle was measured at different time points. Error bars indicate standard deviations (SD), n=16. (C) Root phenotype of 4-week-old seedlings grown in solution culture. Scale bar = 5 cm. (D) Expression level of *OsPIN2* in 7-d-old WT, *lra1* mutant and complementation lines (C1-C3).

Figure S3

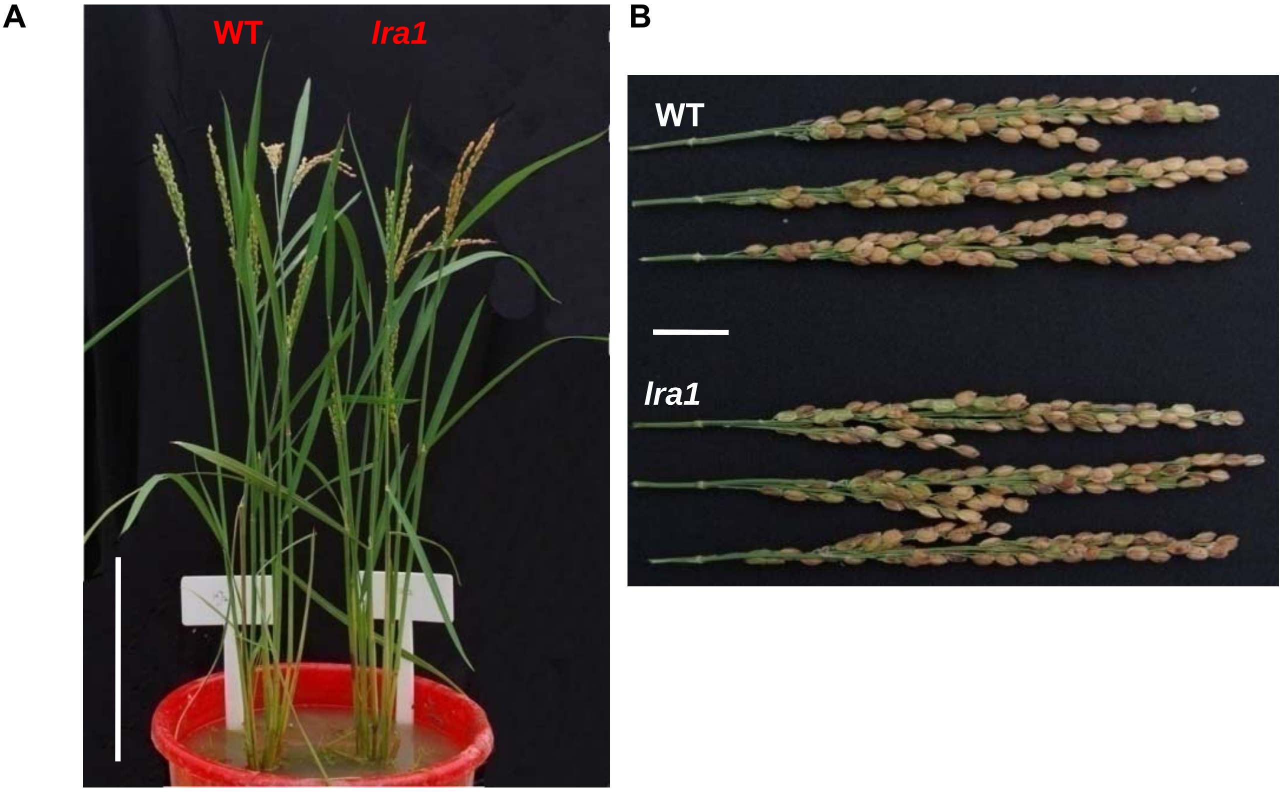

Figure S3. Phenotype of WT and *lra1* grown in soil pots.

(A) Phenotype of 50-d-old WT (left) and *lra1* (right). Scale bar = 20 cm

(B) The panicles of WT and *lra1*. Scale bar = 2 cm

Figure S4

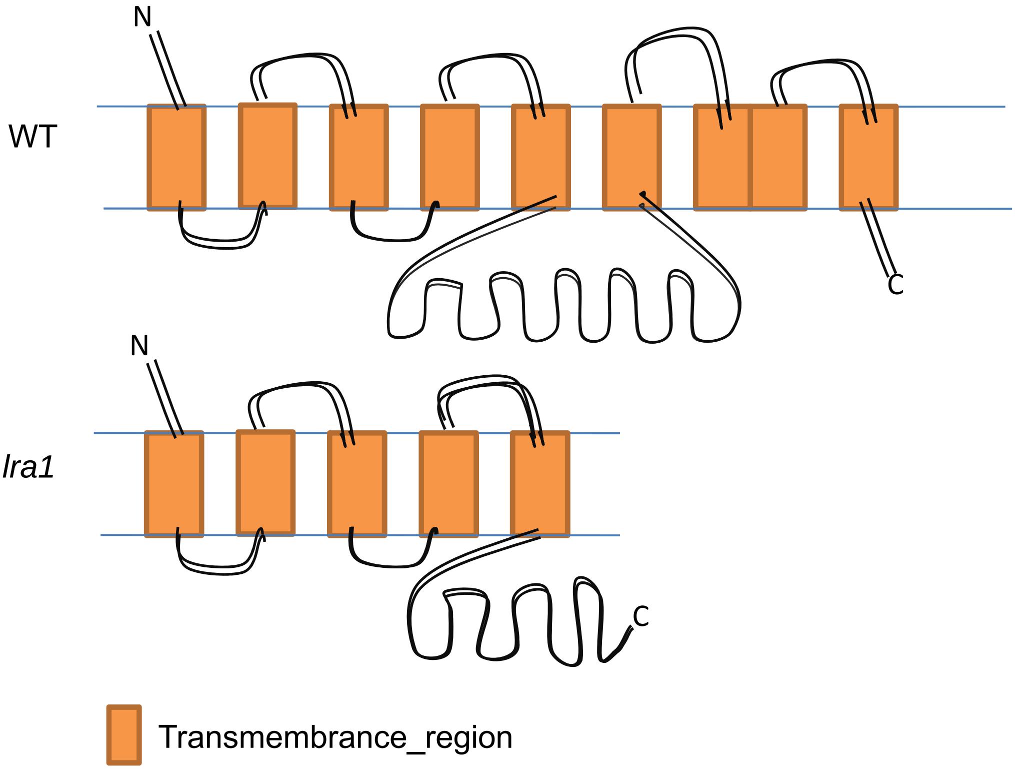

Figure S4. Predicted transmembrane topology models of OsPIN2 protein in WT and *lra1* mutant. InterPro (http://www.ebi.ac.uk/interpro/search/sequence-search) was used to predict the topology.

Figure S5

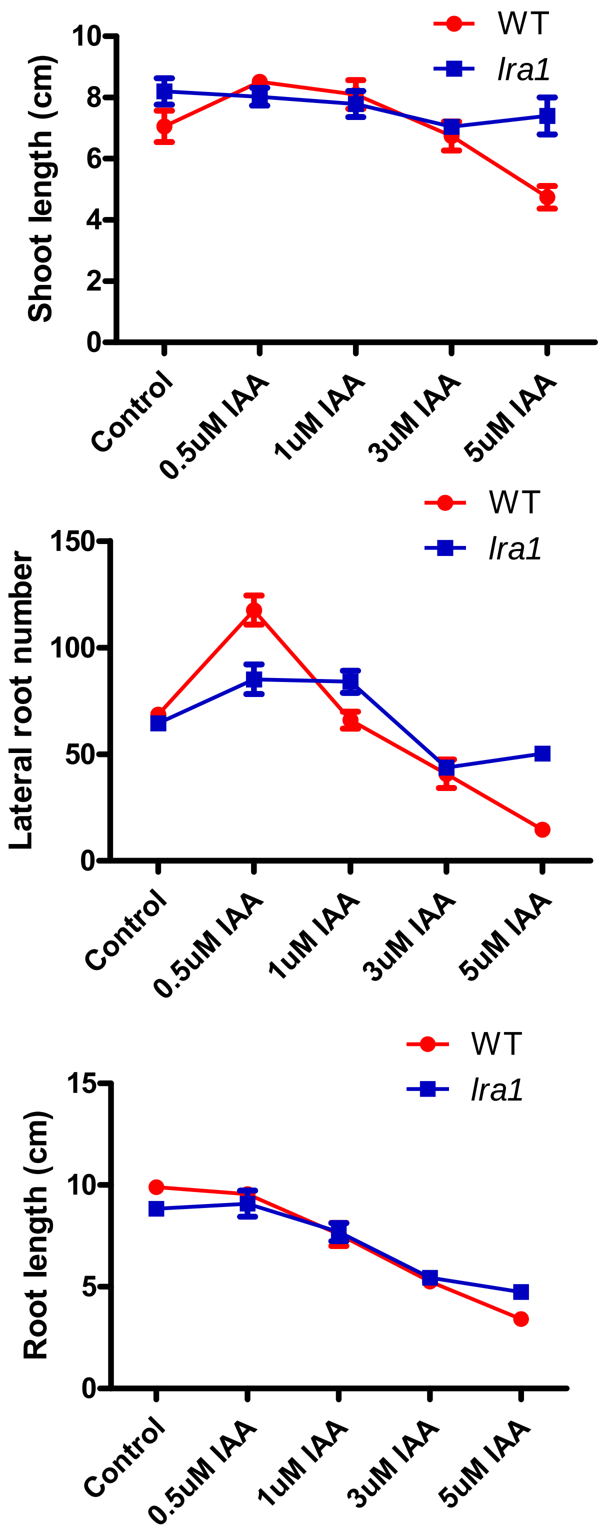

Figure S5. The phenotypic data of the WT and *lra1* seedlings treated with different concentration IAA.

Figure S6

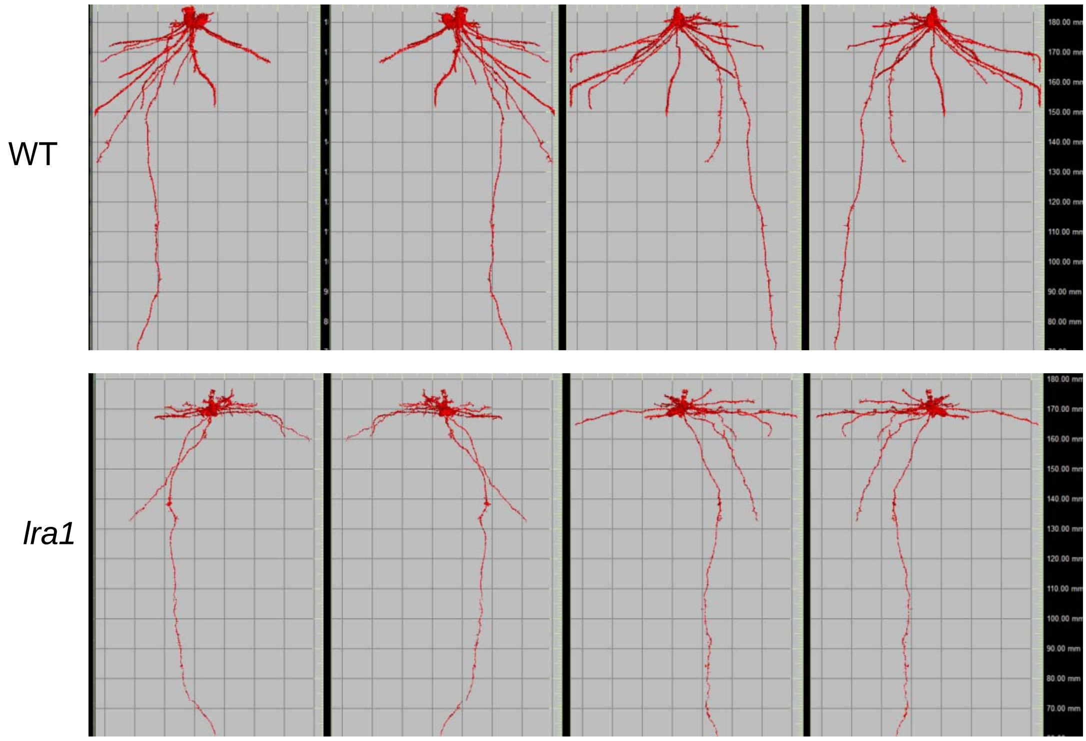

Figure S6. 3D visualization of rice roots segmented from soil viewed from different angles at 21 days after germination.

Figure S7

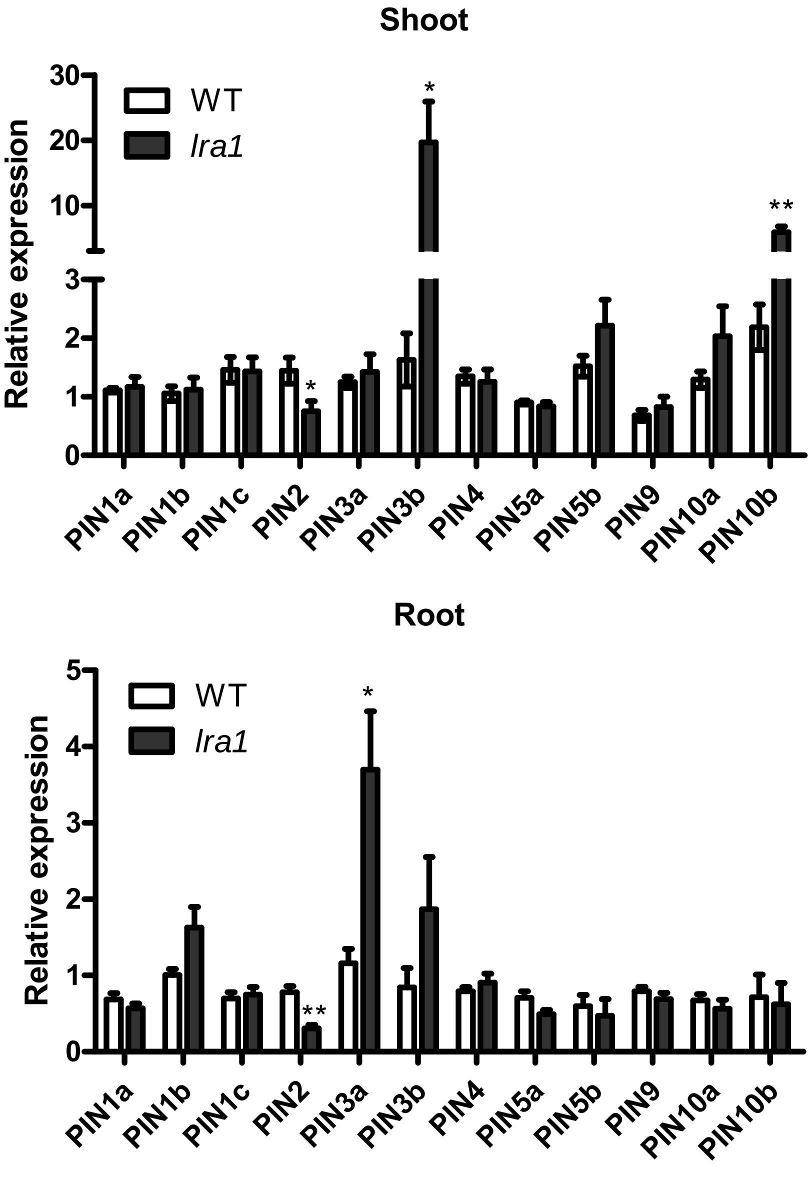

Figure S7. QRT-PCR analysis of PIN family genes expression in shoot and root of WT and *lra1* mutant. Data are means ±SE of 3 replicates , * indicates statistical significance by Student's *t*-test (* $P<0.05$,** $P<0.01$).

Table S1. Primers used in the study.

Name	Sequences (5'-3')
**Primers for identifying the mutant point**	
OsPIN2-F1	GGGGCTCAAACTCCAACTCCAACAA
OsPIN2-R1	TGATCAGATCGTGAACTCAAGTGTA
Primers for CAPS	
CAPS-F	GAGATCGAGGACGGGCTGAA
CAPS-R	GCCCTTGCCCTGAAGATGA
**Primers for complementation**	
OsPIN2-infusion-F1 (*Eco*R I)	CCATGATTACGAATTCCGTTGCTTCCCTGGACATAG
OsPIN2-infusion-R1 (*Hin*d III)	GGCCAGTGCCAAGCTTACGGCTGAACTCTTCATACTCA
**Primers for subcelluar localization**	
eGFP-F(*Kpn* I)	GGGGTACCATGGTGAGCAAGGGCGA
eGFP-R(*Bam*H I)	CGGGATCCCTTGTACAGCTCGT
OsPIN2-cDNA-F(*Xba* I)	GCTCTAGAGGGGTGAGTGGCAGTGTCAC
OsPIN2-cDNA-R(*Sal* I)	ACGCGTCGACTCTTGCCATGCACTGTGGAAAC
OsPIN2-infusion-F1 (*Eco*R I)	CCATGATTACGAATTCCGTTGCTTCCCTGGACATAG
OsPIN2-infusion-R2 (*Kpn* I)	GCCCTTGCTCACCATGGTACCTTTGGGAGTGGCGCCGC
**Primers for tissue expression pattern**	
proOsPIN2-F(*Sal* I)	GCGTCGACACAAATCAGCTGCGAAACGA
proOsPIN2-R(*Bam*H I)	CGGGATCCCGCGCCGGCGACGGT
**Primers for identifying the function in Arabidopsis**	
proAtPIN2-infusion-F3 (*Eco*R I)	TATGACCATGATTACGAATTCCCGTGAATAGAAAG AGGTAACTG
proAtPIN2-infusion-R3 (*Kpn* I)	TCTAGAGGATCCCCGGGTACCTTTGATTTACTTTT TCCGGC
OsPIN2-cDNA-F (*Kpn* I)	GGGGTACCATGATCACCGGACGCGACA
OsPIN2-cDNA-R (*Kpn* I)	GGGGTACCTCCTACTTGATCTCATTTCCCTATT
**Primers for qRT-PCR**	
OsACTIN-RT-F	CAACACCCCTGCTATGTACG
OsACTIN-RT-R	CATCACCAGAGTCCAACACAA
OsPIN1a-RT-F	GACGAGCGTGATGACCCG
OsPIN1a-RT-R	ATGGCCGGCATCTCGAAGTT
OsPIN1b-RT-F	TCAGGTTCCTCGTGGGTC
OsPIN1b-RT-R	ACGGCTGTGCTCAGAATG
OsPIN1c-RT-F	ATCCGCAACCCCAACACC
OsPIN1c-RT-R	AGCGCCATGAACAGCCCGA
OsPIN2-RT2-F	CAACACCTACTCCAGCCTC
OsPIN2-RT2-R	TGGACCAGTCAAGAACCTC
OsPIN3a-RT-F	TCAATCGCCATCGGACTC
OsPIN3a-RT-R	AAAAATTACCGCTGTGCT
OsPIN3b-RT-F	CAGGACGAGCTAGCGAAGCT
OsPIN3b-RT-R	GATTGGCATCGTGATGTGGA
OsPIN4-RT-F	CCTTTGTGTTTGCCAAGGAG
OsPIN4-RT-R	GTGATGGGGAGAGCAATCAG
OsPIN5a-RT-F	GGGGCTGGTGCTAAAGTTC
OsPIN5a-RT-R	GATGTGATGGATTGAGGTAGGG
OsPIN5b-RT-F	CTGCACCTCGCCATCATA
OsPIN5b-RT-R	ATATAACCGCCGTGCTGAG

OsPIN9-RT-F	TTCTGATAGGCCCGGTTGT
OsPIN9-RT-R	CGTACACAAATGATGTCACTGC
OsPIN10a-RT-F	TGATGCTCTTCCTCTTCGAGT
OsPIN10a-RT-R	GACGTGCAGGGACACGAT
OsPIN10b-RT-F	CGAGCTAGCGAAGCTGGA
OsPIN10b-RT-R	CCTTCGTCGTCGTAGTCACC
AtACTIN-RT-F	CATCCTCCGTCTTGACCTTGC
AtACTIN-RT-R	CAAACGAGGGCTGGAACAAG
AtPIN2-RT-F	CCTTGCTTGGTCCCTTGTCT
AtPIN2-RT-R	ATCGCAAACCCTGCTACTGA
**Primers for identifying the transgenic Arabidopsis lines**	
AtPIN2-F (At-F)	TTTGTTCAAATTAACGGACCG
AtPIN2-R (At-R)	AAAAACCTAAGAGTTTTGGAAGTG
LBb1	GCGTGGACCGCTTGCTGCAACT
proAtPIN2-F (At-F1)	GTTTGCTCACTTTCTTCGTT
OsPIN2-R (Os-R)	CAGCGTGGACAGCGAGAAGA

Table S2. Sample properties and scanning settings for X-ray μCT.

**Materials**	**Rice (*Oryza sativa*)**
Experiment conditions	Pot experiment
Soil	Sterilised Kettering Loam
Plant age (weeks)	3
Cylingder internal diameter (cm)	8
Cylingder material	PVC
Number of plants per cylinder	1
Height of scanned part of root system (cm)	18
Height of analyzed part of root system (cm)	18
Voxel size (μm)	60
Current (μA)	180
Voltage (kV)	180
Number of images per subscan	2160
Filtering	0.1 mm copper
Scanning time per plant (min)	75
Scanning parts per plant	2

Table S3. Agronomic traits of wild type (WT) and *lra1* in solution culture (n=10)

**Genotype**	**WT**	***lra1***
Plant height (cm)	80.86 ± 9.80	86.07 ±6.95
Root length (cm)	24.46 ±1.86	25.94±2.63
Tiller number per plant	4.00 ±0.65	3.77 ±0.75
Panicle length (cm)	16.87 ± 0.93	16.55 ± 1.20
Grain weigh (g/panicle)	1.94 ± 0.20	2.00 ± 0.21
Seed setting rate (%)	0.88 ± 0.04	0.89 ± 0.06
Grain number per plant	84.78 ± 12.82	83.59 ± 10.37
Grain length (mm)	6.28	6.58
Grain width (mm)	3.42	3.35
Length/width	1.84	1.97

Data are mean ±SD.

Table S4. Agronomic traits of wild type (WT) and *lra1* in soil pot experiment (n=3)

**Genotype**	**WT**	***lra1***
Plant height (cm)	74.33 ±2.52	63.67 ± 2.08**
Total tiller number per plant	20.00 ± 4.24	23.00 ± 4.24
Effective tiller number per plant	14.50 ± 3.53	17.00 ± 2.82
Panicle length (cm)	13.60 ± 0.85	11.56 ± 1.29
Grain weigh (g/panicle)	1.60 ± 0.14	1.43 ± 0.27
Seed setting rate (%)	0.80 ± 0.11	0.88 ± 0.01
Grain number per plant	83.40 ± 9.10	76.25 ± 5.56
Grain length (mm)	6.50	6.18
Grain width (mm)	3.50	3.27
Length/width	1.86	1.89

Data are mean ±SD. The asterisk indicates statistical significance between WT and *lra1* by Student's *t*-test (**, $P<0.01$).

Table S5. SNPs within OsPIN2 (LOC_Os06g44970.1) in different rice varieties.

SNP ID	Chr	Position	Major Allele	Minor Allele	Variation	Effect	Frequency of major allele in Indica (%)	Frequency of major allele in Japonica (%)	Frequency of minor allele in Indica (%)	Frequency of minor allele in Japonica (%)
sf0627200245	chr06	27200245	C	T	C->T	Synonymous	99.5	100	0.5	0
sf0627200254	chr06	27200254	A	C	C->A	Synonymous	98.25	0.6	1.75	99.4
sf0627200716	chr06	27200716	C	T	C->T	Synonymous	99.62	100	0.38	0
sf0627201163	chr06	27201163	A	G	G->A	Non-Synonymous	98.25	0.8	1.75	99.2
sf0627201294	chr06	27201294	G	A	G->A	Synonymous	84.86	100	15.14	0
sf0627201316	chr06	27201316	G	T	T->G	Non-Synonymous	98.12	0.8	1.88	99.2
sf0627201577	chr06	27201577	T	G	T->G	INTRON	99.62	100	0.38	0
sf0627201597	chr06	27201597	C	T	C->T	INTRON	99.87	100	0.13	0
sf0627201599	chr06	27201599	T	G	G->T	INTRON	98.12	0.6	1.88	99.4
sf0627201725	chr06	27201725	G	A	G->A	INTRON	99.87	99.8	0.13	0.2
sf0627201744	chr06	27201744	T	C	C->T	INTRON	98.25	0.8	1.75	99.2
sf0627201768	chr06	27201768	C	G	G->C	INTRON	98.25	0.8	1.75	99.2
sf0627201796	chr06	27201796	G	T	T->G	INTRON	98.12	0.8	1.88	99.2
sf0627201808	chr06	27201808	G	T	T->G	INTRON	98	0.8	2	99.2
sf0627201835	chr06	27201835	T	A	A->T	INTRON	98	0.6	2	99.4
sf0627201891	chr06	27201891	T	A	T->A	INTRON	20.25	99.8	79.75	0.2
sf0627201892	chr06	27201892	A	T	T->A	INTRON	97.87	0.6	2.13	99.4
sf0627202559	chr06	27202559	T	C	C->T	INTRON	98.12	0.8	1.88	99.2
sf0627203511	chr06	27203511	A	T	A->T	UTR_3_PRIME	99.62	100	0.38	0
sf0627203558	chr06	27203558	G	A	A->G	UTR_3_PRIME	98.25	0.6	1.75	99.4
sf0627203667	chr06	27203667	C	T	T->C	UTR_3_PRIME	98.25	0.6	1.75	99.4

The SNPs were analyzed by using RiceVarMap (http://ricevarmap.ncpgr.cn/). 799 Indica varieties and 497 japonica varieties were used. The two none synonymous SNPs were shaded. Chr, Chromosome; Position, chromosome position;
